# Complex Dorsal Dislocation of the Metacarpophalangeal Joint of the Fifth Finger Treated by Open Reduction With Volar Approach: A Case Report

**DOI:** 10.7759/cureus.84603

**Published:** 2025-05-22

**Authors:** Mohammed Boubcheur, Abdelilah Rhoul, Samir Bensalah, Hicham Yacoubi

**Affiliations:** 1 Orthopaedics and Traumatology, Centre Hospitalier Universitaire Mohammed VI, Oujda, MAR; 2 Faculty of Medicine and Pharmacy, Mohammed First University, Oujda, MAR; 3 Physical Medicine and Rehabilitation, Centre Hospitalier Universitaire Mohammed VI, Oujda, MAR; 4 Orthopaedics, Centre Hospitalier Universitaire Mohammed VI, Oujda, MAR

**Keywords:** fifth digit, hand trauma management, kaplan's lesion, little finger, metacarpophalangeal joint dislocation, volar approach

## Abstract

Complex dorsal dislocations of the metacarpophalangeal (MCP) joint are rare, with involvement of the fifth finger being exceedingly uncommon. These injuries typically result from hyperextension forces that entrap the volar plate, precluding successful closed reduction and necessitating surgical intervention. In this report, we present the case of a 20-year-old male patient who sustained multiple injuries, including craniofacial trauma and an open dorsal dislocation of the left fifth MCP joint, following a road traffic accident. Initial attempts at closed reduction under local anesthesia were unsuccessful, prompting surgical consultation. The patient underwent open reduction via a volar approach, which involved a vertical incision adjacent to the metacarpal head. Intraoperative findings revealed a ruptured volar plate with an attached sesamoid bone, as well as displaced soft tissue structures, including the natatory and superficial transverse ligaments. Gentle retraction and manipulation using a Freer elevator facilitated the reduction of the proximal phalanx and allowed for reconstruction of the volar plate. Postoperative management included brief splinting followed by hand rehabilitation. Radiographic evaluation confirmed maintained reduction without re-dislocation, and the patient ultimately regained nearly full range of motion with no long-term functional deficits or pain. This case highlights the challenges in managing complex MCP dislocations and underscores the importance of prompt surgical intervention. A review of the current literature is provided, discussing the merits and drawbacks of various open reduction techniques, with an emphasis on the volar approach as a reliable option for achieving favorable outcomes in such rare injuries.

## Introduction

Hand trauma is one of the most frequent orthopedic emergencies, with interphalangeal joint sprains as well as phalangeal and metacarpal fractures being commonly observed. In contrast, dislocations of the metacarpophalangeal (MCP) joint occur much less often. The complex dislocation of the MCP joint is a rare injury [1]. It is an irreducible dislocation of the MCP joint of the finger, usually caused by the entrapment of the volar plate, and is considered a severe injury that often needs surgical reduction [2]. A dislocation is defined as simple if it can be easily reduced without the need for open surgery, and as complex if open reduction is required [3]. The index finger is most commonly involved, followed by the thumb, which is described by Kaplan in 1957, who shared his observation in two cases where the reduction of dislocation was not feasible by external maneuver and therefore required surgical reduction [4]. Complex dislocation of the fifth finger’s MCP joint is an exceedingly rare injury; only a handful of cases have been documented [3]. In this report, we present an additional case and detail the clinical features, pathological findings, and management strategy employed.

## Case presentation

A 20-year-old male student was the victim of a road traffic accident resulting in craniofacial trauma and deformity. He sustained a hyperextension injury of his left non-dominant fifth finger, leading to pain and functional impairment. The patient was transferred to the trauma department, where he was managed. The diagnosis was multiple facial fractures and open dislocation of the fifth MCP joint, based on clinical and radiological examinations. All attempts by the emergency physician to perform a closed reduction under local anesthesia failed, and orthopedic consultation was sought.

**Figure 1:**
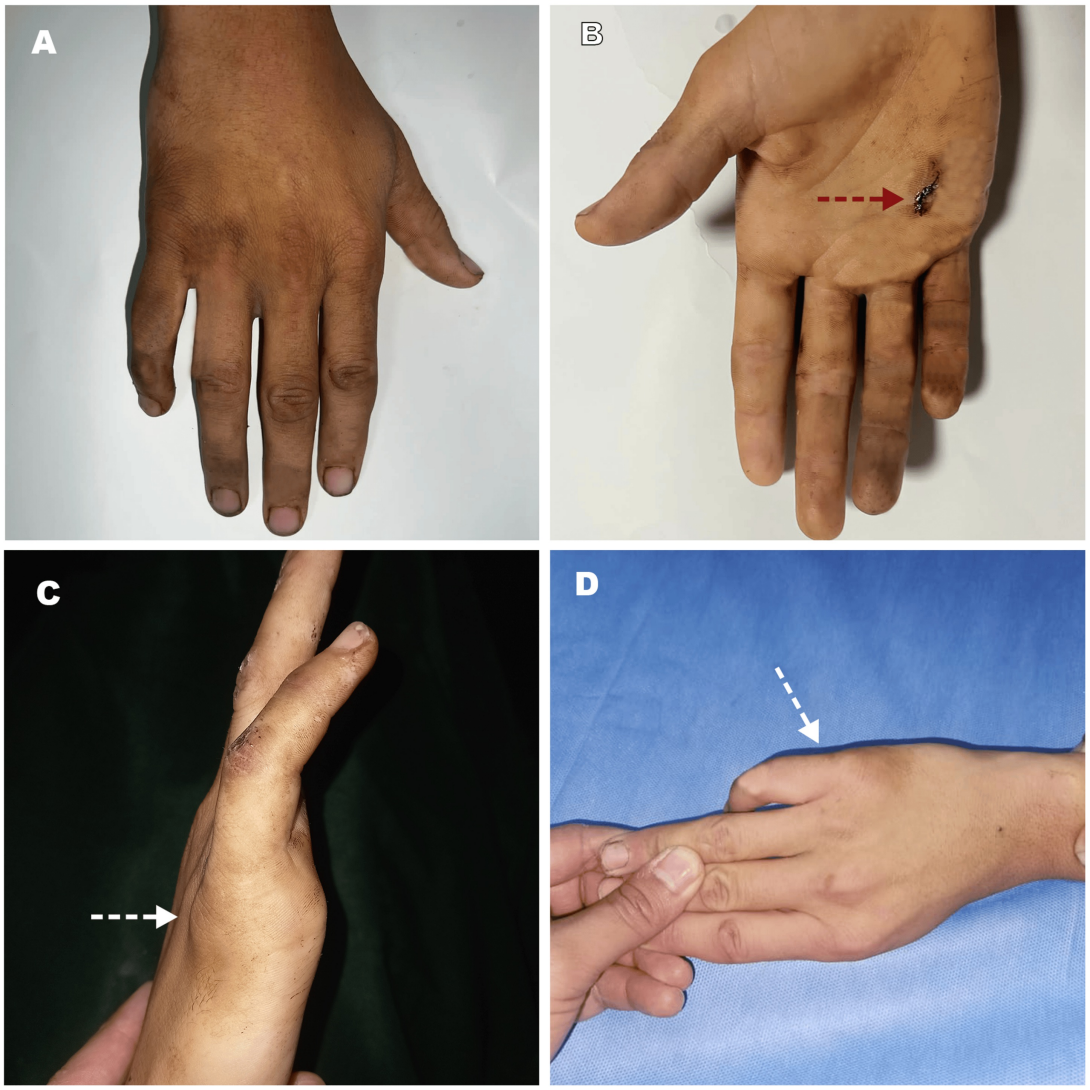
Clinical aspect of the complex metacarpophalangeal joint dislocation of the right little finger (A) Dorsal view of the hand; (B) Frontal view with the palmar wound (red dashed arrow); (C) Flessum (white dashed arrow) and edema on the dorsum of the right fifth finger; (D) Dorsal view indicating dorsal swelling of the metacarpophalangeal joint of the little finger

On admission, three hours after the accident, he was conscious, with facial swelling and multiple ecchymoses without hemodynamic or respiratory distress. Physical examination revealed a 0.5 cm palmar wound over the head of the fifth metacarpal and the proximal phalanx of the right fifth finger in extension position, with mild swelling of the dorsal fifth MCP joint (Figure 1); all manipulations of the digit were painful, with a marked palpable prominence in the palm. There was no evidence of digital neurovascular abnormalities.

**Figure 2:**
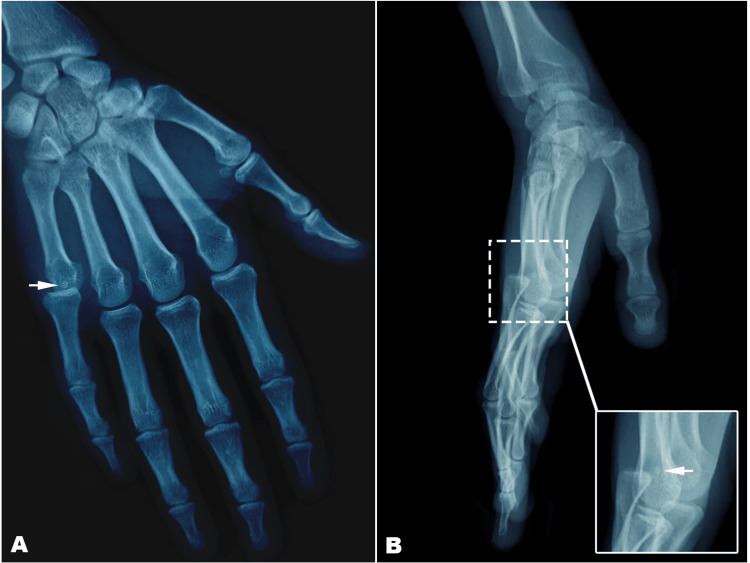
Preoperative X-rays of the right hand. (A) Anteroposterior view of the hand showed dorsal complex metacarpophalangeal joint dislocation of the small finger; (B) Lateral view of the hand showing the pure dislocation with no osteochondral fragment; sesamoid bone entrapment within joint (small white arrow).

The patient underwent a head and neck CT scan, which confirmed the multiple fractures of the facial bones; the cerebral window was normal. Radiographs showed a dorsal dislocation of the metacarpophalangeal joint of the small finger with entrapment of the sesamoid bone (Figure 2). The staff decision was to proceed with open reduction without further attempts at closed reduction.

**Figure 3:**
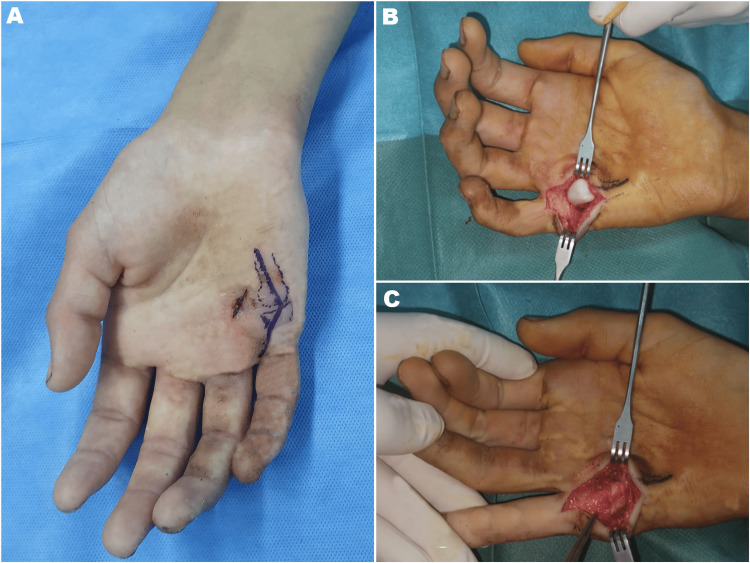
Intraoperative photographs showing the volar approach (A) Volar approach of the metacarpophalangeal joint; (B) Volar view reveals a dislocated metacarpophalangeal joint, with the entrapped volar plate clearly positioned adjacent to the metacarpal head; (C) Aspect after reduction of the dislocation and repair of the damaged tissue

**Figure 4:**
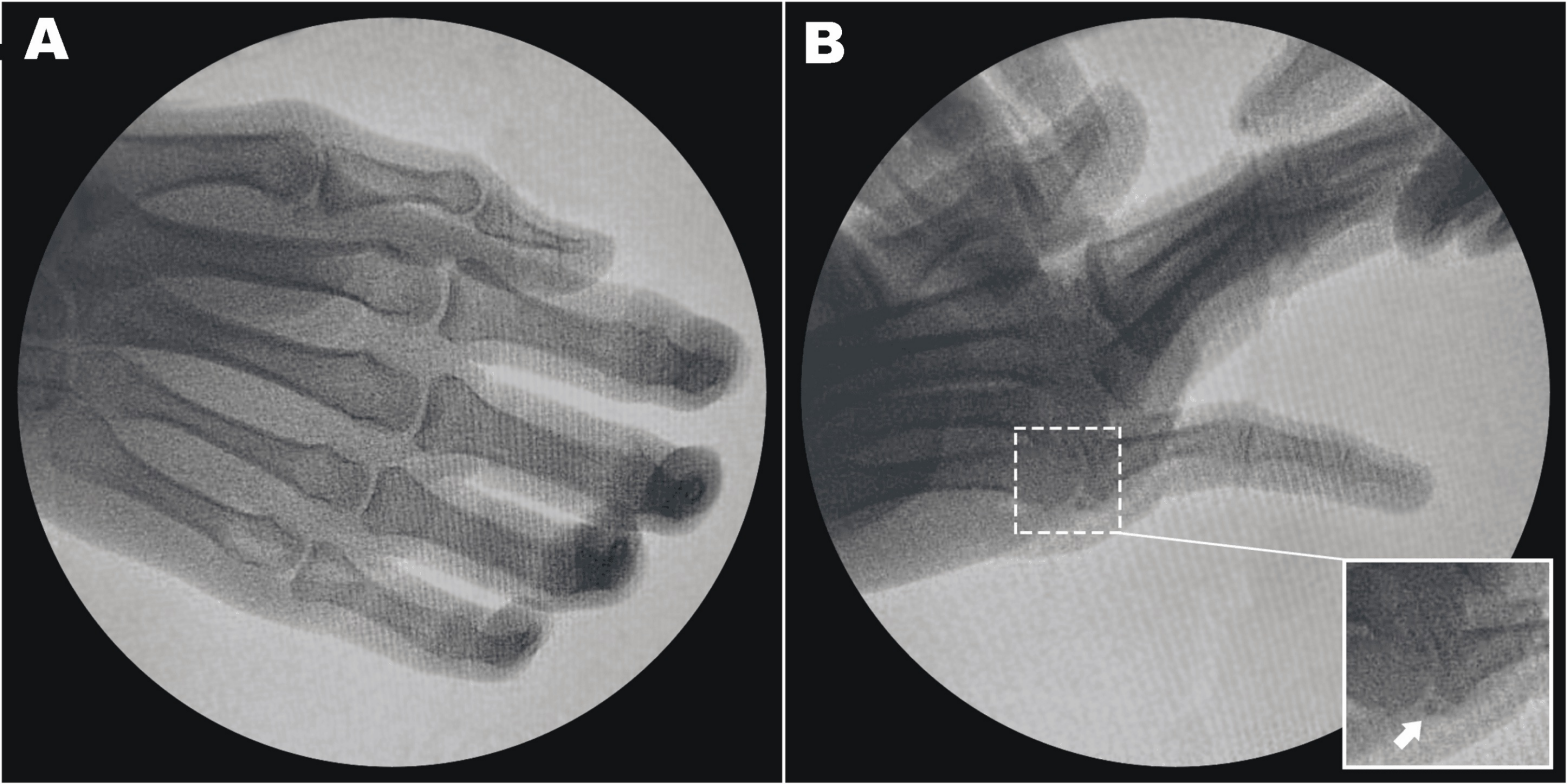
Intraoperative fluoroscopy. (A) Anteroposterior view shows the reduction of the dislocation; (B) Oblique view showing the reduced MCP joint, with a sesamoid bone in place (white arrow).

Two 2 cm-long vertical volar incisions were made adjacent to the metacarpal head along the horizontal wound. Elevating the distal edge of the incision revealed the natatory ligament, while lifting the proximal edge exposed the superficial transverse ligament. At the ulnar border of the fifth metacarpal head, the muscle bellies of the abductor digiti minimi (ADM) and flexor digiti minimi brevis (FDM) were identified, and the little finger’s flexor tendon was visible at the radial border (Figure 3). No fractures, tendon damage, or neurovascular damage were noted upon further examination. The ruptured volar plate was found within the dislocated space, with a sesamoid bone attached to its proximal end. With the wrist flexed and the surrounding structures gently retracted, the proximal phalanx and its attached volar plate were carefully reduced using a Freer elevator. After removing the entrapped, ruptured volar plate, the proximal phalanx was realigned and the volar plate reconstructed, resulting in the sesamoid bone being buried beneath it. Figure 4 shows the intraoperative fluoroscopy images. The repair of the volar plate and subcutaneous tissues was completed using Vicryl 3-0 sutures (Ethicon, Inc., Raritan, New Jersey, United States), and the skin was closed with nylon sutures. An ulnar groove splint was then applied to minimize joint movement for 14 days, after which hand rehabilitation began. Postoperative x-rays confirmed that no re-dislocation had occurred.

**Figure 5:**
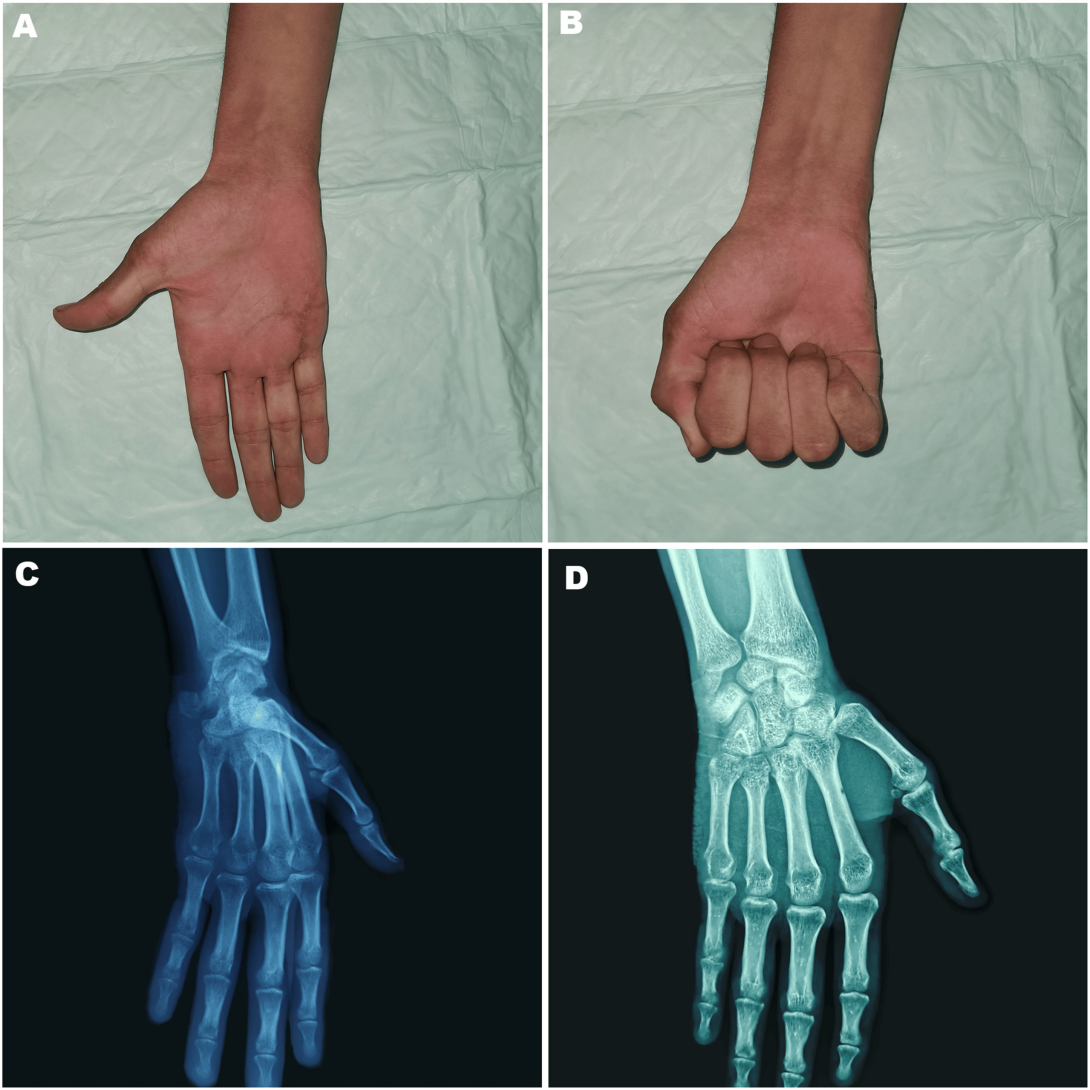
Clinical photographs and X-ray images at the one-year follow-up (A-B) Range of motion was assessed showing (A) extension, (B) flexion; (C-D) No redislocation and no evidence of arthritis

At the eight-week follow-up, the patient demonstrated active range of motion. The MCP joint exhibited 10° of hyperextension and 75° of flexion; the proximal interphalangeal joint reached 0° in extension and 90° in flexion; and the distal interphalangeal joint achieved 0° in extension with 90° in flexion. After one year, the patient reported being pain-free and had regained nearly full range of motion in the little finger MCP joint. On X-ray, the reduction was maintained, and there is no sign of joint arthritis (Figure 5).

## Discussion

Complex dorsal MCP joint dislocations are usually caused by hyperextension injuries, during which the volar plate becomes trapped between the metacarpal head and the proximal phalanx; this entrapment prevents closed reduction and is commonly referred to as Kaplan’s lesion [5].

Kaplan’s lesions result from the rupture of the volar plate, a thick, sturdy tissue in a hyperextended MCP joint. As a consequence, these lesions are uncommon, with roughly 50% of cases also presenting a fracture of the MCP head. Kaplan’s lesions most frequently affect the index finger, followed by the thumb and little finger [6].

Dorsal dislocations are more frequently encountered and typically require surgical reduction when they are complex. In contrast, volar dislocations are rare and can involve the entrapment of the dorsal capsule, which complicates closed management [7].

The pathoanatomy involves the dislocation of the MCP head, which becomes “buttonholed” within the surrounding soft tissues. Kaplan’s original description details this process: the fibrocartilaginous plate is avulsed from its weakest attachment at the volar aspect of the second metacarpal neck, the flexor tendon and pre-tendinous band are displaced ulnarly, while the lumbricals shift radially relative to the metacarpal head [8]. The fibrocartilaginous plate then moves dorsally over the metacarpal head and becomes wedged between the base of the proximal phalanx and the metacarpal head. Furthermore, the laterally displaced collateral ligaments lock the phalanx in an abnormal dorsal position. Distally, the natatory ligament lies dorsal to the metacarpal head along with the volar plate, and proximally, the superficial transverse ligament extends volarly across the metacarpal neck [9].

In the index finger, the flexor tendons shift ulnarly while the lumbrical moves radially. In the little finger, the common tendon of the abductor digiti minimi and flexor digiti minimi is displaced ulnarly, with both the flexor tendons and lumbrical moving radially, the natatory ligament is displaced dorsally, and the superficial transverse metacarpal ligament shifts proximally [6].

Open surgical reduction techniques for MCP joints include the traditional volar and dorsal approaches as well as arthroscopic and percutaneous methods. The volar and dorsal approaches remain the most commonly used and well-documented methods [10,11]. It is important to note that each approach has its own advantages and disadvantages, and there is ongoing debate over which is superior [12]. The dorsal approach offers easier access to the entrapped volar plate injury and poses a lower risk of neurovascular bundle injury. However, this technique requires splitting the extensor mechanism and the dorsal joint capsule, does not permit repair of the volar plate, and complicates the management of dislocations older than three weeks due to potential entrapment of the flexor and lumbrical tendons [13]. In contrast, the volar approach, as described by Kaplan, allows for easier reduction of entrapments involving structures other than the volar plate and enables volar plate repair, though it increases the risk of injuring the neurovascular bundles, which may be displaced anteriorly. Additionally, Pereira et al. have described a lateral approach that provides access to both dorsal and palmar structures and reduces the risk of scarring, making it valuable in cases requiring fixation of osteochondral fragments [14]. Nonetheless, this method carries a higher risk of neurological injury, which limits its use. Regardless of the chosen technique, early joint reduction is critical to minimize the risk of growth arrest, osteonecrosis, or joint stiffness.

Many complications in patients with complex dorsal MPJ dislocations are associated with delayed diagnosis, accompanying osteochondral fractures, multiple closed reduction attempts, traumatic open reduction, or extended immobilization. Long-term complications include early degenerative arthritis and osteonecrosis of the metacarpal head [15]. The literature highlights joint stiffness as the most frequent complication of this injury, potentially arising from the initial soft tissue trauma, prolonged immobilization, even as brief as 10 days, or osteochondral fracture, leading to subsequent degenerative changes. Less common complications include pain and premature epiphyseal closure resulting in metacarpal shortening [16].

Despite the potential for complications, when complex dorsal MPJ dislocation is treated on the day of injury using either dorsal or volar open reduction techniques, patients can ultimately achieve excellent results and satisfactory long-term outcomes, characterized by no functional deficits, no pain, and minimal arthritis [17].

## Conclusions

Complex dorsal dislocations of the MCP joint typically arise from hyperextension injuries and cannot be reduced by closed maneuvers. The primary obstacle is the volar plate, which becomes displaced between the dorsum of the metacarpal head and the volar base of the proximal phalanx. Kaplan’s lesions are clinically rare because the volar aspect of the MCP joint is reinforced by stronger adjacent structures. Most lesions occur in the index finger and are exceptionally uncommon in the little finger.

Since these lesions are rare, no standard treatment protocol has been established. Various approaches may be applied depending on the specific traumatic condition and the surgeon’s preference. Currently, the volar approach to the MCP joint is considered more reliable for managing dorsal dislocations because it facilitates the removal of interposed soft tissue and the repair of the sagittal bands, although it does have limitations in cases involving dislocated fractures and carries a risk of vasculo-nervous injury. Loss of motion is the most common complication of this injury, especially when the diagnosis is delayed. Nevertheless, complex dorsal MCP joint dislocations treated with open reduction techniques on the day of injury can lead to satisfactory long-term outcomes.
